# Predicting response and toxicity to PD-1 inhibition using serum autoantibodies identified from immuno-mass spectrometry

**DOI:** 10.12688/f1000research.22715.1

**Published:** 2020-05-07

**Authors:** Milena Music, Marco Iafolla, Antoninus Soosaipillai, Ihor Batruch, Ioannis Prassas, Melania Pintilie, Aaron R. Hansen, Philippe L. Bedard, Stephanie Lheureux, Anna Spreafico, Albiruni Abdul Razak, Lillian L. Siu, Eleftherios P. Diamandis

**Affiliations:** 1 Department of Laboratory Medicine and Pathobiology, University of Toronto, Toronto, ON, Canada; 2 Division of Medical Oncology and Hematology, University Health Network, Canada, Toronto, ON, Canada; 3 Department of Pathology and Laboratory Medicine, Mount Sinai Hospital, Toronto, Toronto, ON, Canada; 4 Department of Biostatistics, Princess Margaret Cancer Centre, University Health Network, Canada, Toronto, ON, Canada; 5 Department of Clinical Biochemistry, University Health Network, Canada, Toronto, ON, Canada; 6 Lunenfeld-Tanenbaum Research Institute, Mount Sinai Hospital, Toronto, Toronto, ON, Canada

**Keywords:** programmed cell death protein 1, immune checkpoint blockade, pembrolizumab, response, predictive biomarkers, autoantibodies, anti-thyroglobulin antibody, anti-thyroid peroxidase antibody, hypothyroidism, immune-related adverse events

## Abstract

**Background: **Validated biomarkers are needed to identify patients at increased risk of immune-related adverse events (irAEs) to immune checkpoint blockade (ICB). Antibodies directed against endogenous antigens can change after exposure to ICB.

**Methods:** Patients with different solid tumors stratified into cohorts received pembrolizumab every 3 weeks in a Phase II trial (INSPIRE study). Blood samples were collected prior to first pembrolizumab exposure (baseline) and approximately 7 weeks (pre-cycle 3) into treatment. In a discovery analysis, autoantibody target immuno-mass spectrometry was performed in baseline and pre-cycle 3 pooled sera of 24 INSPIRE patients based on clinical benefit (CBR) and irAEs.

**Results:** Thyroglobulin (Tg) and thyroid peroxidase (TPO) were identified as the candidate autoantibody targets. In the overall cohort of 78 patients, the frequency of CBR and irAEs from pembrolizumab was 31% and 24%, respectively. Patients with an anti-Tg titer increase ≥1.5x from baseline to pre-cycle 3 were more likely to have irAEs relative to patients without this increase in unadjusted, cohort adjusted, and multivariable models (OR=17.4, 95% CI 1.8–173.8, p=0.015). Similarly, patients with an anti-TPO titer ≥ 1.5x from baseline to pre-cycle 3 were more likely to have irAEs relative to patients without the increase in unadjusted and cohort adjusted (OR=6.1, 95% CI 1.1–32.7, p=0.035) models. Further, the cohort adjusted analysis showed patients with anti-Tg titer greater than median (10.0 IU/mL) at pre-cycle 3 were more likely to have irAEs (OR=4.7, 95% CI 1.2–17.8, p=0.024). Patients with pre-cycle 3 anti-TPO titers greater than median (10.0 IU/mL) had a significant difference in overall survival (23.8 vs 11.5 months; HR=1.8, 95% CI 1.0–3.2, p=0.05).

**Conclusions: **Patient increase ≥1.5x of anti-Tg and anti-TPO titers from baseline to pre-cycle 3 were associated with irAEs from pembrolizumab, and patients with elevated pre-cycle 3 anti-TPO titers had an improvement in overall survival.

## List of abbreviations

ABC, ammonium bicarbonate; CBR, clinical benefit rate; CI, confidence intervals; CR, complete response; CTLA-4, cytotoxic T-lymphocyte-associated antigen 4; FDR, false discovery rate; HR, hazard ratios; ICB, immune checkpoint blockade; irAE, immune related adverse event; MPS, modified percent score; NSCLC, non-small cell lung cancer; OR, odds ratios; OS, overall survival; PD-1, programmed cell death protein 1; PFS, progression-free survival; PR, partial response; SD, stable disease; Tg, thyroglobulin; TPO, thyroid peroxidase.

## Introduction

Despite the ability of immune checkpoint blockade (ICB) to produce enduring responses in several advanced malignancies ^1, 2^, there are several limitations. Monotherapy response rates for inhibitors of programmed cell death protein-1 or its ligand (anti-PD-1/L1) across solid tumors are generally modest at approximately 20–30%, and much lower in some tumor types ^3, 4^. All-grade and high-grade toxicities with PD-1/L1 approach 40% and 12%, respectively ^5– 8^. Hence, there is an unmet need for predictive biomarkers to enable patient selection. Although numerous markers have already been tested for their ability to predict response to ICB ^1^, many require molecular and immune profiling from either invasive biopsy or archival tumor specimen ^9^. Similarly, validated biomarkers that can identify patients who develop immune-related adverse events (irAEs) to ICB are lacking.

Antigen spreading is the phenomenon of *in situ* damaged cancer cells releasing intracellular antigens into the surrounding environment ^10, 11^. Although tumorigenesis distorts the native structure of these antigens ^12^, which in turn elicit a humoral immune response that produces tumor associated antigen autoantibodies ^13– 16^, several studies have investigated the biomarker potential of autoantibodies targeting wild-type proteins using large human proteome arrays in patients treated with ICB. For instance, castrate resistant prostate cancer patients responding to ipilimumab demonstrate an increase in autoantibodies targeting intracellular proteins (such as MPG, PAK6 and DLX1) relative to non-responders ^17^. Melanoma patients who developed severe irAEs from the combination of intralesional *bacillus Calmette-Guérin* injection and ipilimumab were found to have a significant increase in autoantibodies directed against self-antigens that preceded the clinical diagnosis of toxicity ^18^. Pre-treatment autoantibody profiles in melanoma patients treated with anti-PD-1, anti-CTLA-4 or a combination of both agents were unique to those who developed severe irAEs ^19^. Humoral immunity may be increased via PD-1 inhibition through direct interaction with PD-1 receptors on B-cells ^20^ and/or non-cytotoxic T-cell dependent processes ^21^.

Current attempts to characterize serum autoantibodies are limited by analytic constraints inherent to complex biological samples, such as blood or lysate, that are fraught with low sensitivity ^22, 23^. This study attempts to overcome this analytic limitation by typifying the serum autoantibodies using a modified version of the established Serologic Proteome Analysis assay ^24– 26^ that incorporates protein G purification of serum autoantibodies and tandem mass spectrometric identification of the antibody targets.

We hypothesize that the magnitude of autoantibody production is a measure of response and/or toxicity to ICB. Our primary objective is to isolate candidate serum autoantibodies from advanced solid tumor patients receiving pembrolizumab (anti-PD-1) monotherapy on a prospective phase II clinical trial and correlate their titers with clinical outcomes.

## Methods

### Patients and clinical outcomes

The single-centre investigator-initiated biomarker phase II clinical trial called INSPIRE ( INvestigator-initiated Phase II Study of Pembrolizumab Immunological Response Evaluation; ClinicalTrials.gov ID NCT02644369, registered on 31 December 2015) was designed to analyze dynamic changes in genomic, proteomic and immunologic landscapes in patients treated with pembrolizumab (anti-PD-1 antibody) monotherapy. Between March 21, 2016 to May 9, 2018, patients were prospectively enrolled into one of the following five cohorts: squamous cell cancer of the head and neck, triple negative breast cancer, epithelial ovarian cancer, malignant melanoma (cutaneous and non-cutaneous) or mixed solid tumors. Approximately 20 patients were enrolled into each cohort; the INSPIRE study was purely exploratory and no formal sample size was required. Pertinent inclusion criteria: age ≥18 years; ECOG 0-1; incurable histologically proven locally advanced or metastatic solid malignancy without further standard treatment options (with the exception of melanoma); measurable disease based on RECIST 1.1 ^27^; and adequate organ functions. Pertinent exclusion criteria: prior anti-PD-1/L1/L2 agents (prior anti-CLTA4 and T-cell co-stimulatory agents allowed); autoimmune disease; immunodeficiency or immunosuppressive medications exceeding physiologic corticosteroid replacement; leptomeningeal disease; or <4 weeks of stable central nervous system metastases.

All patients received monotherapy pembrolizumab (anti-PD-1 antibody) 200 mg IV every 3 weeks, were clinically assessed with comprehensive blood work every 3 weeks and received restaging CT scans every 9 weeks. Clinical benefit rate (CBR) was defined *a priori* as RECIST 1.1 complete response (CR) or partial response (PR), or stable disease (SD) for ≥6 cycles of pembrolizumab. Toxicity was defined *a priori* as ≥Grade 2 CTCAE 4.03 ^28^ irAE with at least possible attribution to pembrolizumab of which the investigator asserted the adverse event to be autoimmune in causality from pembrolizumab exposure. Grade 2 or higher grade irAEs are considered clinically significant ^29^. Only the highest grade of a specific toxicity event was recorded for each patient; the same toxicity event was recorded more than once per patient if the irAE re-occurred on a separate occasion (i.e. not a re-flare during immunosuppression taper). Both response and toxicity data were annotated by INSPIRE data coordinators and then verified by one author (MI). The causality of irAE to pembrolizumab was reviewed by MI and LLS.

The Research Ethics Board located at Princess Margaret Cancer Centre, University Health Network (Toronto, Canada) reviewed and approved this study (#15-9828). All patients provided written informed patient consent prior to study enrollment. The study was carried out in accordance with the Declaration of Helsinki.

### Sample collection and storage

Blood samples for this analysis were drawn within 28 days prior to first pembrolizumab exposure (baseline) and 7 weeks into treatment with pembrolizumab (pre-cycle 3). Upon collection of blood samples in Vacutainer® SST™ tubes, the blood was allowed to clot for 30–60 minutes, and the tubes were then centrifuged at 1,200 *g* for 10 minutes at room temperature. In total, 500 μL of serum was aliquoted into cryogenic vials and frozen at -80°C.

### Discovery analysis for candidate autoantibody identification

In order to optimize autoantibody identification, 24 patients treated on INSPIRE were selected by MI and LLS and placed into one of four groups: 6 patients without CBR or toxicity from pembrolizumab (Group I); 6 patients with toxicity but no CBR (Group II); 6 patients with both CBR and toxicity (Group III); and 6 patients with CBR but no toxicity (Group IV). To minimize bias, performance of the experiments and analysis of the results were conducted by investigators blinded to INSPIRE clinical outcomes.

In total, 12 µL of serum from each of the 6 individual patients in the four Groups (I–IV) were pooled to create mixed pools (72 µL/pool). This was done for the serum samples collected both before and after immunotherapy initiation, resulting in the following eight pooled serum samples: Group I (baseline), Group I (pre-cycle 3), Group II (baseline), Group II (pre-cycle 3), Group III (baseline), Group III (pre-cycle 3), Group IV (baseline) and Group IV (pre-cycle 3).

### Human tissue lysate proteome

Human tissues were obtained from Mount Sinai Hospital (REB# 18-0077-E) and the University Health Network (REB #15-9680) at autopsy or during surgical removal. Tissue samples were stored at -80° C until ready for use. Protein was extracted from a total of 29 human body tissues and 4 human brain-specific tissues for comprehensive proteome coverage (see *Extended data* ^30^). For protein extraction, tissue was pulverized in liquid nitrogen with a mortar and pestle to yield a fine powder. Next, 0.2% RapiGest SF Surfacant (Waters, Milford, MA, USA) in 50 mM ammonium bicarbonate (ABC) was added to further lyse the tissues. The pulverized tissue sample was vortexed every 5–10 min on ice, for 30 min, and then sonicated on ice for 15 sec, three times, to further disrupt the cells. Following sonication, the sample was centrifuged at 15,000 *g* for 20 min at 4°C, to remove debris and insoluble contents, followed by collection of the supernatant. A Pierce BCA Protein Assay (Thermo Fisher Scientific, San Jose, California) was performed on tissue lysate protein extracts for total protein quantification. Equal amounts of each tissue lysate were combined to make a 1.5-mg complex tissue lysate for each immunoprecipitation experiment.

### Autoantibody immuno-mass spectrometry

Details are stated in *Extended data* ^30^. A similar method was recently validated in a proof-of-concept study that included autoantibodies to CUB and zona pellucida-like domain-containing protein 1 and pancreatic secretory granule membrane major glycoprotein 2 in the sera of patients with inflammatory bowel disease ^31^.

### Selection of candidate autoantibodies

The autoantibody protein targets identified before and after pembrolizumab treatment in Groups I through IV were compared. Immunoglobulins, keratins, serum albumin and other non-specific serum-abundant proteins (such as hemoglobin), as well as complement proteins and apolipoproteins that are normally immunoprecipitated as part of immune complexes in the blood ^32^, were excluded. The candidates were chosen based on the *a priori* definition of ≥2-fold peptide number increase for the target protein from pre- to post-treatment serum in patient group II, III or IV (n=6 in each group) and either a ≥4-fold peptide number difference compared to patient group I (n=6) or no identified peptides in group I ^17^. Due to cost and feasibility, only the top candidate and a related protein candidate were further evaluated by electro-chemiluminescence immunoassay.

### Electrochemiluminescence immunoassay for anti-Tg and anti-TPO antibody quantification

Immuno-mass spectrometric analysis of the discovery set showed that thyroglobulin (Tg) was the top candidate autoantibody target that met the pre-defined selection criteria. Thyroid peroxidase (TPO) was chosen as an additional candidate autoantibody target due to its known association with anti-Tg antibody in autoimmune disease. Immuno-mass spectrometric results are available as *Underlying data* ^33^.

All INSPIRE patients with baseline and pre-cycle 3 sera (including those used in the discovery analysis) were analyzed for the candidate autoantibody titers by electrochemiluminescence immunoassays (Elescys Anti-Tg and Elecsys Anti-TPO, Roche Diagnostics, Risch-Rotkreuz, Switzerland) using a Cobas e 411 analyzer. The Cobas e 411 analyzer has an approximate 10% coefficient of variation for both anti-Tg ^34^ and anti-TPO ^35^ titers. Further, the coefficient of biological variation of these antibodies is also approximately 10% ^36^. To be conservative, a minimum 50% increase in titers from baseline to pre-cycle 3 was used to define a significant increase. Based upon instrument limitations, the measurable range of anti-TPO and anti-Tg antibodies were 5.00–600 IU/mL and 10.0–4000 IU/mL, respectively. Patients with anti-TPO titers <5.00 IU/mL and anti-Tg titers < 10.0 IU/mL were arbitrarily assigned 5.00 IU/mL and 10.0 IU/mL, respectively. One patient in the mixed solid tumor cohort had an anti-Tg titer >4000 IU/mL both at baseline and cycle 3; the value of 4000 UI/mL was used for both time points. The median value of anti-Tg titers at both baseline and pre-cycle 3 was 10.0 IU/mL (range: 10.0–4000 IU/mL) and the median value of anti-TPO titers at baseline and pre-cycle 3 was 10.10 IU/mL and 10.70 IU/mL, respectively (range: 5.00–434.70 IU/mL). For simplicity, we defined elevated anti-Tg and anti-TPO when the titer was > 10.0 IU/mL. The investigators conducting all experiments and analysis of the results were blinded to INSPIRE clinical outcomes.

### Statistical analysis of candidate autoantibody targets

The co-primary endpoints were CBR and toxicity as defined above. Patients were dichotomized at baseline and pre-cycle 3 using median titers of candidate autoantibodies, and the change (Δ) in autoantibody titer was defined as a 1.5x increase in titer from baseline to pre-cycle 3 versus stable or decrease in titer (only patients who had pre-cycle 3 titers >10.0 IU/mL were included in the Δ analysis). These groups were analyzed as predictors of CBR and toxicity. Due to the possible influence of toxicity on autoantibody production, patients who developed toxicity before the pre-cycle 3 blood draw were removed from both the CBR and toxicity pre-cycle 3 and Δ analysis. Statistical significance among the categorical variables was evaluated using Fisher’s exact test. Further, the individual patient titers at baseline, pre-cycle 3, and difference in titer (defined as pre-cycle 3 titer minus baseline titer) were explored as continuous variables using the Mann-Whitney test. As with the categorical variable analysis above, patients who developed toxicity before the pre-cycle 3 blood draw were removed from both the CBR and toxicity pre-cycle 3 and difference in titer analysis.

In an effort to reduce confounding in our observational study with a small number of events, propensity scores were created based on a model incorporating age, gender, ethnicity and PD-L1 status. As outlined in the interim INSPIRE trial report ^37^, this study used formalin-fixed paraffin-embedded blocks from screening biopsies. The PD-L1 immunohistochemistry clone 22C3 was applied to 4–5 μm sections mounted on positively charged ProbeOn slides (QualTek, Goleta, CA). QualTek produced a modified proportion score (MPS) denoting the proportion of PD-L1-expressing tumor cells and mononuclear inflammatory cells within tumor nests. Further details are found in *Extended data* ^30^. Conditional logistic regression was applied to test each candidate autoantibody and their change adjusting for strata and the propensity scores. Odds ratios (OR), their 95% confidence intervals (CI) and p-values were thus obtained.

Overall survival (OS) and progression free survival (PFS) times were calculated as the durations between the first infusion with pembrolizumab and death or progression, respectively. When death or progression were not observed the patient was considered censored. The survival percentages and median survival estimates were calculated using Kaplan-Meier method. The Hazard Ratios (HR) and their CIs were calculated within the Cox Proportional-Hazards Model. The association of toxicity with OS and PFS was analyzed using toxicity as a time-dependent covariate. The association between covariates and candidate autoantibodies were investigated using Fisher’s exact test for categorical variables, or Mann-Whitney test or Spearman correlation for continuous variables (where appropriate).

All p-values were two-sided and p-values < 0.05 were considered statistically significant.

All calculations were performed using R 3.4 (The R Foundation for Statistical Computing).

## Results

### Patient characteristics, serum and outcomes

In total, 106 patients were enrolled into INSPIRE and 78 patients were included in this analysis. Among the excluded 28 INSPIRE patients: 2 patients had both baseline and pre-cycle 3 serum blood work but the pre-cycle 3 serum was not processed for autoantibody quantification; 17 patients were taken off trial between cycle 2 and cycle 3; and 9 patients were taken off trial before cycle 2. Deidentified patient outcomes are available as *Underlying data* ^33^.

The 24 patient samples used in the discovery analysis were frozen for a median of 566 days (range 335–835 days) and thawed twice. All 78 patients used in the test set (which included the 24 patients in the discovery analysis) were frozen for a median of 626.5 days (range 257–927 days) and thawed once.

Table 1 summarizes pertinent clinicopathologic information and their association with CBR and toxicity. Gender was approximately evenly distributed (female 55%, male 45%). The median age was 61 years at time of first pembrolizumab infusion, but there was a large range in age (21–82 years). The vast majority of patients were of white ethnicity (n=64, 83%). Head and neck squamous cell carcinoma (n=14, 18%) comprised the major tumor type. Median PD-L1 MPS were 1% (range: 0 – 100%). Unadjusted univariate analysis suggested the response rate was different between the cohorts (p=0.011) and the patients who had CBR tended to have a higher percent of PD-L1 MPS in comparison to those without CBR (medians 1.5% vs 0%, p=0.029).

**Table 1.** Characteristics of the 78 patients analyzed in this trial stratified by those who did or did not develop CBR or toxicity to pembrolizumab. Of the entire INSPIRE cohort (n=106), 28 patients did not have paired baseline and pre-cycle 3 serum for analysis and thus were excluded.

		No. (%) of patients			No. (%) of patients		
Covariates		With CBR (n=24)	Without CBR (n=54)	p-value	With toxicity (n=19)	Without toxicity (n=59)	p-value
Sex, female		12 (50)	31 (57)	0.62 ^a^	7 (37)	36 (61)	0.11 ^a^
Ethnicity, white		20 (83)	44 (83)	>0.99 ^a^	18 (95)	46 (79)	0.17 ^a^
Age at time of first pembrolizumab infusion, median (range), years		62 (34–82)	58 (21–78)	0.38 ^b^	58 (28–73)	61 (21–82)	0.95 ^b^
Cohort	A: HNSCC	3 (13)	11 (20)	**0.011** ^a^	3 (16)	11 (19)	0.15 ^a^
	B: TNBC	1 (4)	11 (20)		1 (5)	11 (19)	
	C: HGSOC	2 (8)	10 (19)		3 (16)	9 (15)	
	D: Melanoma	8 (33)	3 (6)		6 (32)	5 (9)	
	E: Mixed solid tumor	10 (42)	19 (35)		6 (32)	23 (39)	
PD-L1 MPS positive cells ≥ 1%		16 (67)	24 (44)	0.088 ^a^	11 (58)	29 (49)	0.6 ^a^
% PD-L1 MPS positive cells, median (range)		1.5 (0-100)	0 (0-95)	**0.029** ^b^	2 (0-100)	0 (0-95)	0.12 ^b^

CBR = clinical benefit rate; HGSOC = high-grade serous ovarian carcinoma; HNSCC = head and neck squamous cell carcinoma; MPS = modified percent score; TNBC = triple negative breast cancer. ^a^ P-value calculated by Fisher Exact Test, unadjusted. ^b^ P-value calculated by Mann-Whitney, unadjusted.

**Figure 1.**
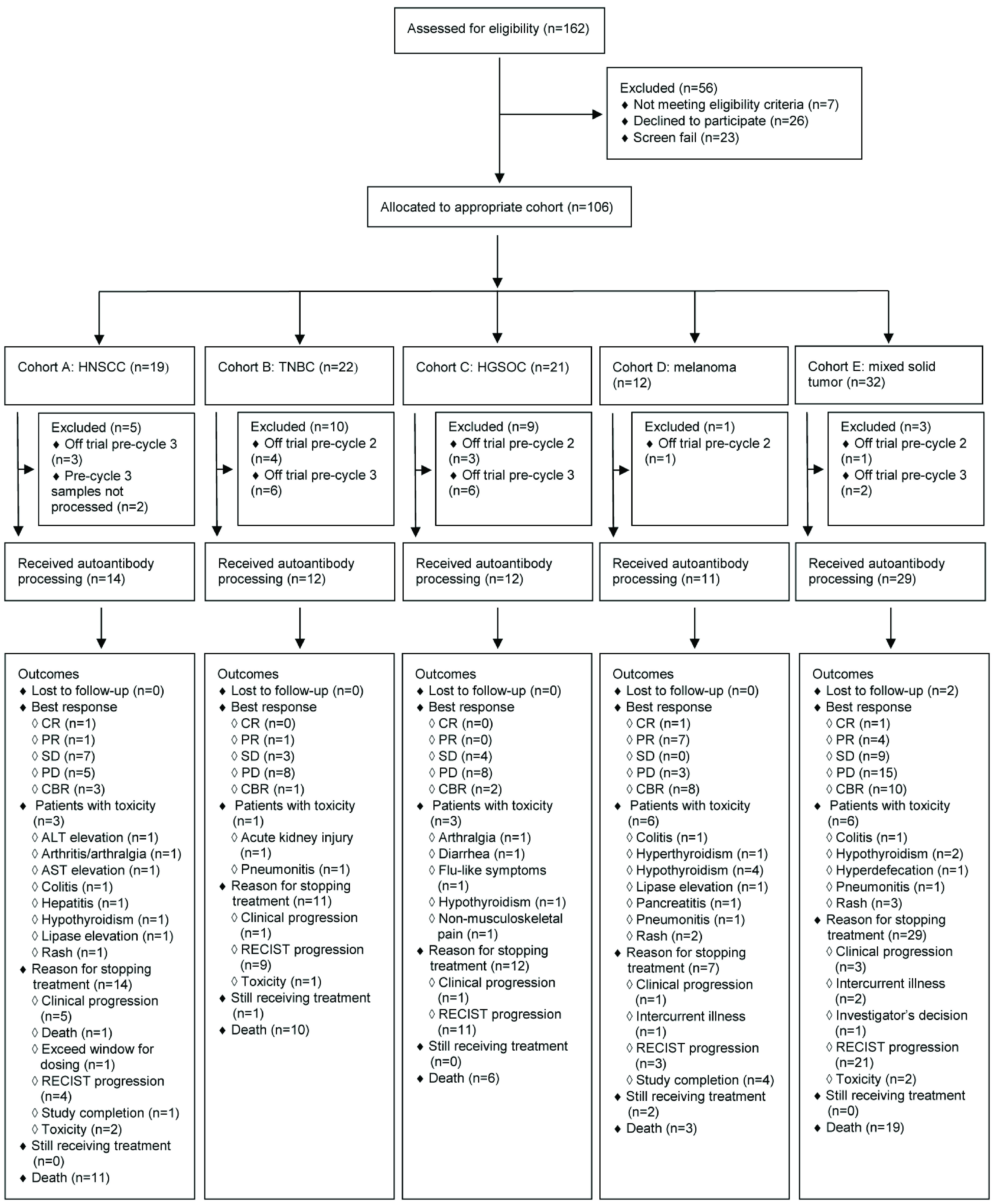
CONSORT diagram displaying all relevant details regarding the 78 INSPIRE patients used in the autoantibody analysis. Note that patients can develop more than one type of toxicity event. HNSCC, head and neck squamous cell carcinoma; TNBC, triple-negative breast cancer; HGSOC, high-grade serous ovarian carcinoma; CR, complete response; PR, partial response; SD, stable disease; PD, progressive disease; CBR, clinical benefit rate; ALT, alanine aminotransferase; AST, aspartate aminotransferase.

The CONSORT diagram in Figure 1 depicts the relevant patient and serum sample flow through this study and clinical outcomes of each cohort. Last clinical outcome update was May 3, 2019; at that time the median follow-up was 2.35 years from date of first pembrolizumab infusion (range: 0.79–2.95 years), two patients (2.6%) were lost to follow up and 49 deaths (63%) occurred. Of the two patients lost to follow up, both developed disease progression as best response and one developed a significant toxicity event; both patients were included in the analysis. The median number of pembrolizumab infusions before coming off trial was five (range: 2–35 infusions). All patients were evaluable. CBR was achieved in 24 patients (31%): CR, PR and SD ≥6 cycles of pembrolizumab occurred in 3 patients (3.9%), 13 patients (17%) and 8 patients (10%), respectively. In total, three patients (3.9%) remained on treatment at time of analysis. RECIST disease progression (n=48, 62%) was the major reason for stopping treatment; five patients (6.4%) completed all 35 pembrolizumab cycles specified in the trial. Toxicity occurred in 19 patients (24%); hypothyroidism (n=8, 10%) was the most common toxicity, and nine patients (12%) developed more than one toxicity. Median number of days from first dose of pembrolizumab to toxicity onset was 105 (range: 1–482) and five patients (6.4%) developed toxicity before pre-cycle 3 blood draw. Toxicity necessitated stopping treatment in five patients (6.4%); of these, Grade 3 colitis (n=2, 2.6%) and Grade 3 pneumonitis (n=2, 2.6%) were the most common events. In total, seven patients (9.0%) developed Grade 3 toxicity, two patients (2.6%) developed more than one Grade 3 event and all remaining toxicity events were Grade 2; there were no Grade 4 or 5 events.

### Discovery analysis and anti-Tg and anti-TPO candidate autoantibodies

**Figure 2.**
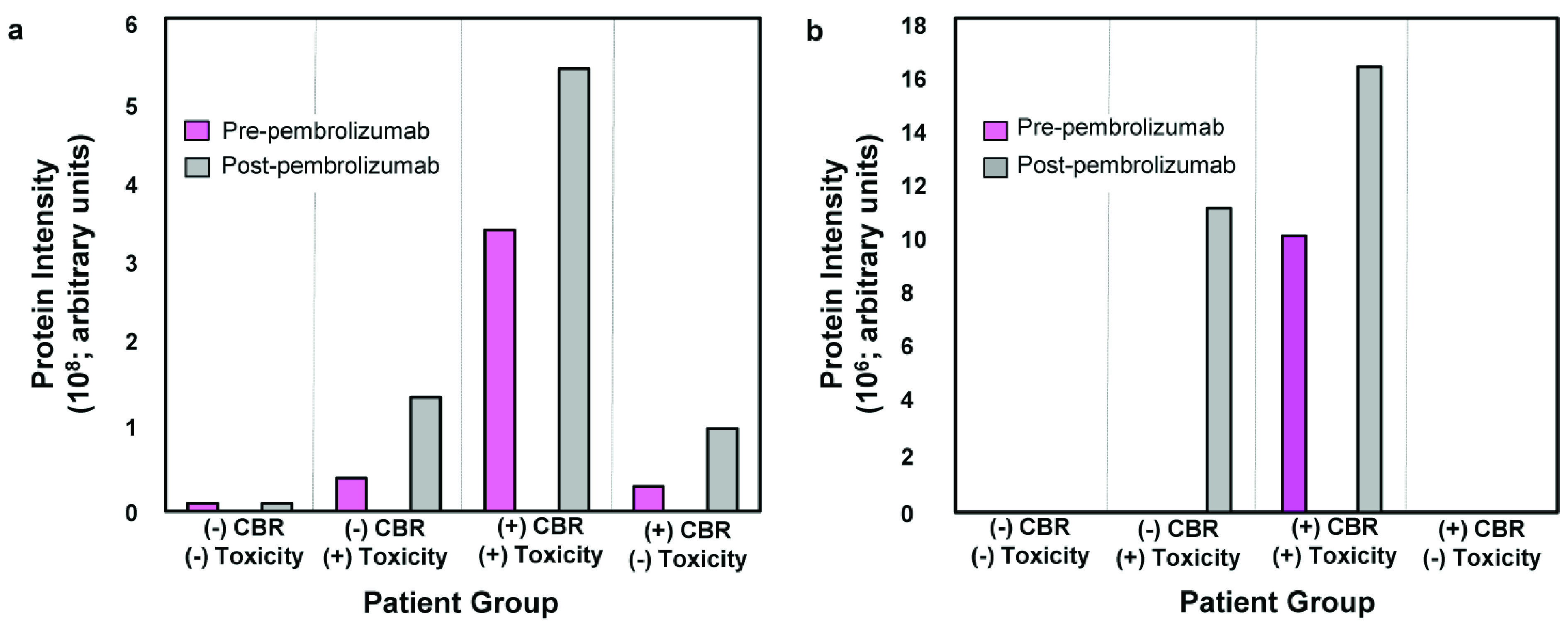
Protein intensities within the pre-specified four INSPIRE groups. The average baseline and pre-cycle 3 thyroglobulin (Tg) (a) and thyroid peroxidase (TPO) (b) protein intensities. We identified Tg and TPO as candidates during autoantibody target selection from pooled patient sera. CBR, clinical benefit rate.

Of the 24 patients used in the discovery pooled serum analysis, melanoma (n=9; 38%) and ovarian cancer (n=5; 21%) comprised the majority. Two patients who were initially assigned to Group IV (+CBR / -toxicity) later developed immune-related hypothyroidism. There were 14 patients with toxicity in this discovery analysis; the most common events were hypothyroidism (n=6, 43%), rash (n=3, 21%) and pneumonitis (n=3, 21%). In total, four patients developed toxicity before the pre-cycle 3 blood draw. The protein intensities of Tg and TPO identified by immuno-mass spectrometry in the four patient group discovery analysis at both baseline and pre-cycle 3 are shown in Figure 2a and Figure 2b, respectively.

Of all 78 patients included in this analysis, at baseline, 20 patients had both elevated anti-Tg and anti-TPO titers, three patients had only elevated anti-Tg titers, and 19 had only elevated anti-TPO titers. At pre-cycle 3, 24 patients had both elevated anti-Tg and anti-TPO titers, two patients had only elevated anti-Tg titers, and 18 had only elevated anti-TPO titers. In total, 10 and eight patients developed a significant Δ in anti-Tg and anti-TPO titers, respectively. No patients developed ≥50% decrease in titer from baseline to pre-cycle 3. De-identified patient autoantibody titres are available as *Underlying data* ^33^.

### Anti-Tg and anti-TPO antibodies as predictive biomarkers

Anti-Tg ( Table 2) and anti-TPO ( Table 3) titers at baseline, pre-cycle 3 and Δ were analyzed for their association with CBR and toxicity. The analysis of the dichotomized values suggested some associations between anti-Tg and toxicity: Δ was significant in unadjusted (p=0.0024), cohort adjusted (OR=23.8, 95% CI 2.6–221.5, p=0.0053), and multivariable models (OR=17.4, 95% CI 1.8–173.8, p=0.015), suggesting an increased risk for toxicity for patients with a pre- anti-Tg cycle 3 titer ≥ 1.5x from baseline. Higher levels of anti-Tg pre-cycle 3 also predicted for higher rate of toxicity when adjusting for cohort (OR=4.7, 95% CI 1.2–17.8, p=0.024). Similarly, anti-TPO Δ was significant for predicting toxicity in patients with ≥1.5x increase in anti-TPO titers from baseline to pre-cycle 3 in unadjusted (p=0.039) and cohort adjusted (OR=6.1, 95% CI 1.1–32.7, p=0.035) models, although significance was lost in multivariable analysis (p=0.078). Anti-Tg titers at baseline and pre-cycle 3 were not significant for predicting toxicity in multivariate analysis, and anti-TPO baseline and pre-cycle 3 titers were not significant for predicting toxicity in any of the analyses. Further, anti-Tg and anti-TPO titers were not associated with CBR at any time point regardless of statistical approach.

**Table 2.** Categorical analysis association of anti-Tg antibodies with CBR and toxicity. Baseline and pre-cycle 3 categories were dichotomized by median titers (10.0 IU/mL) versus > median titers (10.0 IU/mL was anti-Tg titer's lowest limit of instrument detection). Δ was dichotomized using pre-cycle 3 titers ≥ 1.5x baseline titers vs pre-cycle 3 titers < 1.5x baseline titers. Patients who developed toxicity before pre-cycle 3 blood draw (n=5) were removed from both the CBR and toxicity pre-cycle 3 and Δ analysis.

Event	Time point	Method of dichotomization	Unadjusted			Adjusted for cohort		Multivariable ^a^	
			With event – no. (%)	Without event – no. (%)	P value ^b^	OR (95% CI)	P value ^c^	OR (95% CI)	P value ^d^
*CBR*	Baseline	10.0 IU/mL	17 (71)	38 (70)	> 0.99	0.9 (0.3–2.9)	0.83	0.9 (0.2–3.5)	0.91
		> 10.0 IU/mL	7 (29)	16 (30)					
	Pre-cycle 3	10.0 IU/mL	18 (78)	31 (62)	0.19	0.4 (0.1–1.5)	0.17	0.3 (0.09–1.3)	0.12
		> 10.0 IU/mL	5 (22)	19 (38)					
	Δ	Pre-cycle 3 < 1.5x baseline	20 (87)	43 (86)	> 0.99	0.8 (0.1–4.3)	0.75	0.8 (0.1–5.1)	0.79
		Pre-cycle 3 ≥ 1.5x baseline	3 (13)	7 (14)					
*Toxicity*	Baseline	10.0 IU/mL	10 (53)	45 (76)	0.08	2.8 (0.9–8.7)	0.076	2.7 (0.6–11.2)	0.18
		> 10.0 IU/mL	9 (47)	14 (24)					
	Pre-cycle 3	10.0 IU/mL	6 (42.9)	43 (73)	0.055	4.7 (1.2–17.8)	**0.024**	2.6 (0.6–12.3)	0.23
		> 10.0 IU/mL	8 (57)	16 (27)					
	Δ	Pre-cycle 3 < 1.5x baseline	8 (57)	55 (93)	**0.0024**	23.8 (2.6–221.5)	**0.0053**	17.4 (1.8–173.8)	**0.015**
		Pre-cycle 3 ≥ 1.5x baseline	6 (43)	4 (7)					

CBR = clinical benefit rate; CI = confidence intervals; OR = odds ratio. ^a^Determined using propensity scores with cohort as strata and the following covariates: age, gender, ethnicity and PD-L1 status. One patient was removed due to unknown ethnicity. ^b^Calculated using Fisher’s Exact Test. ^c^Based on conditional logistic regression with the cohort as strata. ^d^Based on conditional logistic regression with the cohort as strata and adjusted for the propensity scores.

**Table 3.** Categorical analysis association of anti-TPO antibodies with CBR and toxicity. Baseline and pre-cycle 3 categories were dichotomized by ≤ to median titers (10.0 IU/mL) versus > median titers. Δ was dichotomized using pre-cycle 3 titers ≥ 1.5x baseline titers vs pre-cycle 3 titers < 1.5x baseline titers. Patients who developed toxicity before pre-cycle 3 blood draw (n=5) were removed from both the CBR and toxicity pre-cycle 3 and Δ analysis.

Event	Time point	Method of dichotomization	Unadjusted			Adjusted for cohort		Multivariable ^a^	
			With event – no. (%)	Without event – no. (%)	P value ^b^	OR (95% CI)	P value ^c^	OR (95% CI)	P value ^d^
CBR	Baseline	≤ 10.0 IU/mL	11 (46)	28 (52)	0.81	1.3 (0.5–3.6)	0.65	1.0 (0.3–3.1)	0.98
		> 10.0 IU/mL	13 (54)	26 (48)					
	Pre-cycle 3	≤ 10.0 IU/mL	11 (48)	22 (44)	0.80	1.0 (0.3–2.8)	0.97	1.0 (0.3–3.2)	0.94
		> 10.0 IU/mL	12 (52)	28 (56)					
	Δ	Pre-cycle 3 < 1.5x baseline	21 (91)	44 (88)	> 0.99	0.5 (0.08–3.1)	0.45	0.7 (0.1–4.8)	0.71
		Pre-cycle 3 ≥ 1.5x baseline	2 (9)	6 (12)					
Toxicity	Baseline	≤ 10.0 IU/mL	8 (42)	31 (53)	0.60	1.6 (0.5–4.6)	0.41	1.4 (0.4–4.7)	0.61
		> 10.0 IU/mL	11 (58)	28 (48)					
	Pre-cycle 3	≤ 10.0 IU/mL	4 (29)	29 (49)	0.23	2.7 (0.7–10.3)	0.14	2.3 (0.5–10.2)	0.26
		> 10.0 IU/mL	10 (71)	30 (51)					
	Δ	Pre-cycle 3 < 1.5x baseline	10 (71)	55 (93)	**0.039**	6.1 (1.1–32.7)	**0.035**	5.9 (0.8–42.6)	0.078
		Pre-cycle 3 ≥ 1.5x baseline	4 (29)	4 (7)					

CBR = clinical benefit rate; CI = confidence intervals; OR = odds ratio. ^a^Determined using propensity scores with cohort as strata and the following covariates: age, gender, ethnicity and PD-L1 status. One patient was removed due to unknown ethnicity. ^b^Calculated using Fisher’s Exact Test. ^c^Based on conditional logistic regression with the cohort as strata. ^d^Based on conditional logistic regression with the cohort as strata and adjusted for the propensity scores.

Anti-Tg and anti-TPO titers were also explored as continuous variables for their possible association with CBR and toxicity. The Mann-Whitney test (unadjusted analysis) showed an association of elevated anti-Tg titers with higher risk of toxicity at baseline (p=0.043), pre-cycle 3 (p=0.011) and the difference in titer between these two time points (p=0.001) ( *Extended data* ^30^); significance was lost in cohort adjusted and multivariable models. The same type of association was found with anti-TPO: elevated anti-TPO titers were associated with higher risk of toxicity at pre-cycle 3 in the unadjusted model (p=0.045) and difference in titers between baseline and pre-cycle 3 in the cohort adjusted model (p=0.05), although significance was lost in multivariable analysis ( *Extended data* ^30^). When analyzed as a continuous variable, anti-Tg and anti-TPO were not predictive of CBR ( *Extended data* ^30^).

Prior to trial enrolment, three patients had pre-existing thyroid disease: two patients with hypothyroidism both had elevated anti-TPO titers at baseline and/or pre-cycle 3 and the one patient with thyroid nodules had non-elevated titers; none of these patients developed either CBR or toxicity on trial. Hypothyroidism development during trial (n=8) was more common among: patients with elevated anti-Tg titers at baseline (75% vs 24%, p=0.007), pre-cycle 3 (86% vs 27%, p=0.0044) and increase in titers between these two time points (71% vs 7%, p=0.00026); and in patients with an increase in anti-TPO titer between baseline and pre-cycle 3 (57% vs 7%, p=0.0029). Of the eight patients who developed hypothyroidism, six (75%) had both elevated anti-Tg and anti-TPO antibodies at baseline and pre-cycle 3, one patient (12.5%) developed elevated anti-Tg and anti-TPO titers at pre-cycle 3, and the last patient had non-elevated titers at both time points. Conversely, of the 70 patients who did not develop hypothyroidism, 42 (60%) had elevated anti-Tg and/or anti-TPO titers at baseline and/or pre-cycle 3. There was no statistically significant association of anti-Tg or anti-TPO with the development of non-thyroid toxicities. Of the 11 patients who developed non-thyroidal toxicity, five (46%) had either anti-Tg or anti-TPO titers elevated at baseline or pre-cycle 3.

### Anti-Tg and anti-TPO antibodies as prognostic biomarkers

**Figure 3.**
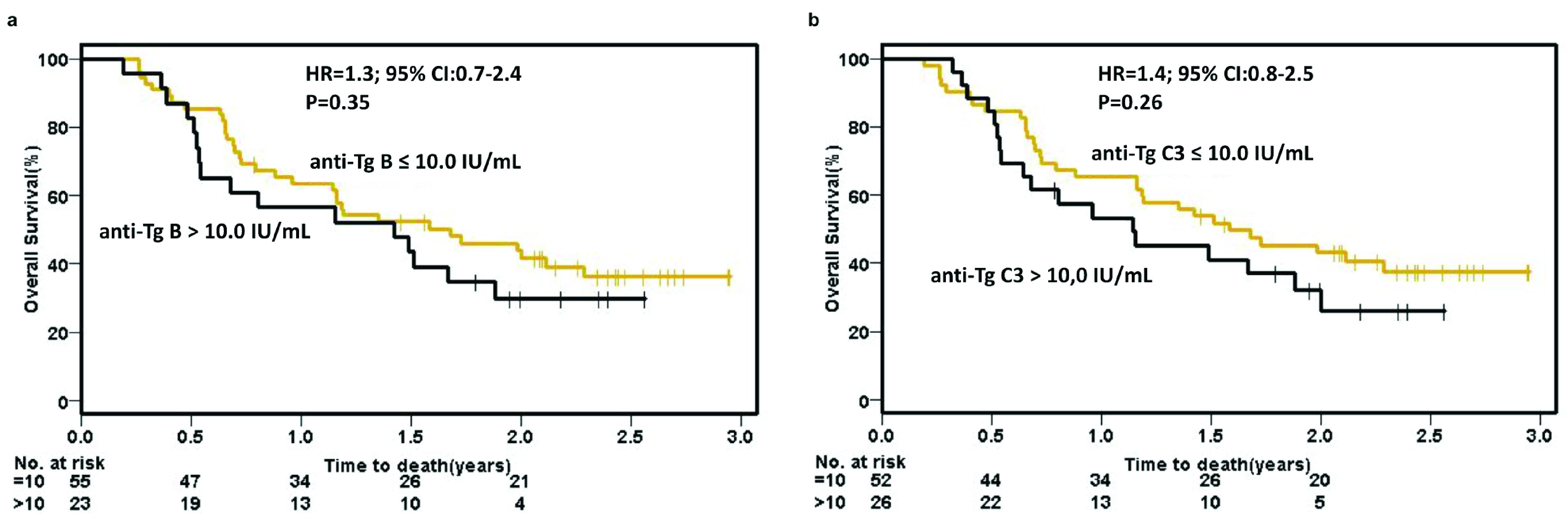
Kaplan-Meier overall survival analysis using anti-Tg titers. Patients were dichotomized based upon anti-thyroglobulin (Tg) titers greater than, or equal to, median (10.0 IU/mL) at ( **a**) baseline and ( **b**) pre-cycle 3. B, baseline; C3, pre-cycle 3; CI, confidence interval; HR, hazard ratio.

**Figure 4.**
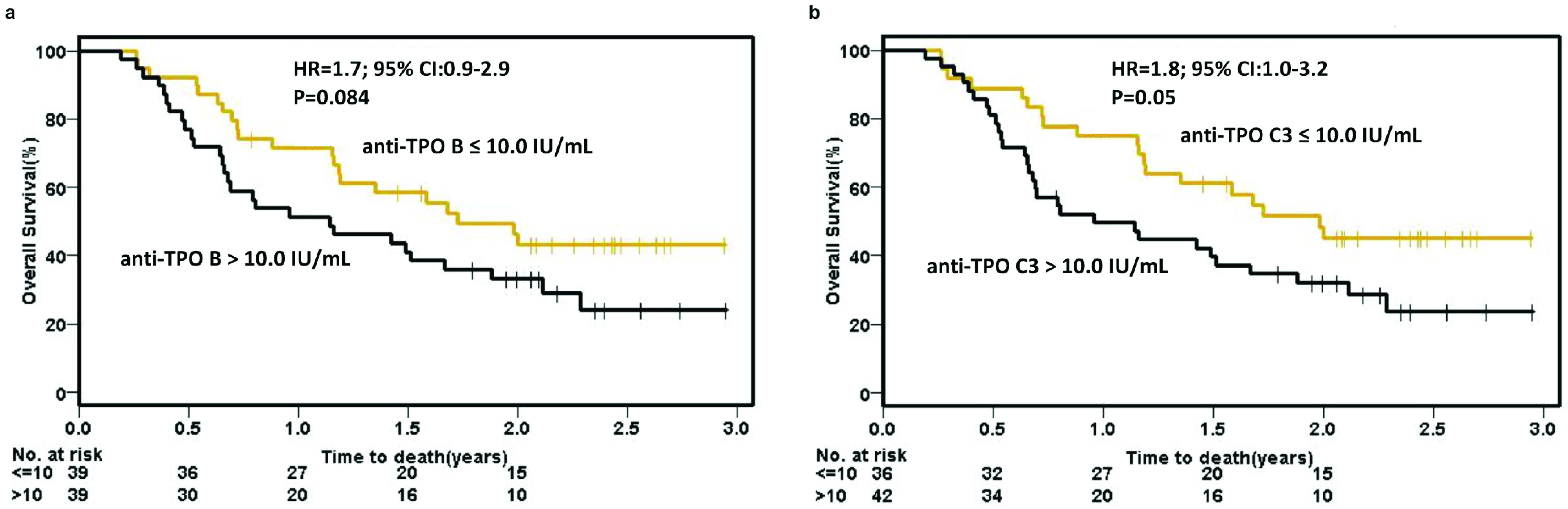
Kaplan-Meier overall survival analysis using anti-thyroid peroxidase (TPO) titers. Patients were dichotomized based upon anti-TPO levels greater than median (10.0 IU/mL) or ≤ median at ( **a**) baseline and ( **b**) pre-cycle 3. P-values are unadjusted and calculated using the Wald test. B, baseline; C3, pre-cycle 3; CI, confidence interval; HR, hazard ratio.

Elevated baseline anti-Tg or anti-TPO titers did not show a significant difference in OS ( Figure 3a and Figure 4a, respectively) or PFS (see *Extended data* ^30^). Although elevated pre-cycle 3 anti-Tg titers were not associated with OS ( Figure 3b), patients with elevated pre-cycle 3 anti-TPO titers did have a significant difference in OS (23.8 months vs 11.5 months; HR=1.8, 95% CI 1.0–3.2, p=0.05) ( Figure 4b). Pre-cycle 3 anti-Tg and anti-TPO titers were not associated with PFS (see *Extended data* ^30^). Finally, anti-Tg and anti-TPO Δ was not significant for OS and PFS (see *Extended data* ^30^).

Toxicity analysed as a time-dependent covariate was not associated with OS (p=0.11) or PFS (p=0.47).

### Association of anti-Tg and anti-TPO antibodies with standard prognostic variables

Associations between the candidate autoantibodies were explored as both categorical and continuous variables with the confounding covariates: age, gender, ethnicity and PD-L1 status. White ethnicity was associated with higher anti-Tg titers at baseline (continuous: p=0.048) and pre-cycle 3 (categorical: p=0.05; continuous: p=0.031) (see *Extended data* ^30^). The remaining covariates did not exhibit any association with the autoantibodies (see *Extended data* ^30^). The Spearman correlation coefficients calculated between the autoantibodies with age and PD-L1 as continuous variables were very small ranging between -0.167 and 0.14, suggesting lack of association between these variables (see *Extended data* ^30^). **

## Discussion

To our knowledge, this is the first study to perform an extensive autoantibody analysis in the pre- and post-pembrolizumab sera of patients with mixed solid tumors, and then determine the association of candidate autoantibodies with clinical benefit and toxicity. Our study suggested that patients with an increase in anti-Tg or anti-TPO titers from baseline to pre-cycle 3 are associated with significant toxicity in unadjusted and cohort-adjusted models, and the anti-Tg increase is also significant for toxicity in multivariable models. Hypothyroidism was observed in 10% in our cohort, which is consistent with the 6.5–7.9% incidence reported in an ICB meta-analysis ^38^, and accounted for 42% of all our toxicity events. Nearly half of our patients with non-thyroid toxicity had elevated anti-Tg and/or anti-TPO titers; however, we were unable to demonstrate an association of these antibodies with non-thyroid toxicity. A recent retrospective analysis of non-small cell lung cancer (NSCLC) patients treated with anti-PD-1 monotherapy demonstrated similar results: pre-treatment anti-Tg and/or anti-TPO antibodies were associated with the development of immune-related hypothyroidism, but not other irAEs ^39^. While antibodies for multiple autoimmune illnesses can develop simultaneously ^40– 42^, our analysis only chose the candidate with the largest number of identified peptides and a related candidate, and thus any non-thyroidal autoantibodies that may have been present were not analyzed. Future work assessing a larger spectrum of autoantibodies to create a biomarker signature will aid in addressing this issue.

Herein we found an association between elevated pre-cycle 3 anti-TPO antibodies with OS. This is in keeping with the NSCLC study that showed baseline autoantibodies (including anti-Tg and anti-TPO) were associated with improved PFS and disease control rate during treatment with anti-PD-1 monotherapy ^39^. However, given our lack of association between anti-TPO with CBR and PFS, and baseline and Δ anti-TPO with OS, caution must be used in interpreting this result. Future trials enriching for specific disease sites will help elucidate the role autoantibodies may play as a prognostic and predictive biomarker to ICB.

The association of irAEs with increased survival or response to ICB has been found in melanoma ^43^ and NSCLC ^44^ studies using statistical methods to prevent bias from the time-dependence of both predictor and outcome variables ^45, 46^. The analysis of toxicity as a time-dependent covariate did not corroborate this principle in our prospective trial of multiple histologies. If benefit is most likely procured from patients with toxicity events, then astute monitoring and early recognition of irAEs is paramount to control these toxicity events and prevent early treatment discontinuation.

Not surprisingly, our association of baseline and on treatment anti-Tg or anti-TPO antibodies with the development of hypothyroidism is supported by other anti-PD-1 studies ^20, 21, 39, 47^. Interestingly, it appears cancer patients harbor higher rates of pre-existent autoantibodies: our pre-treatment combined anti-Tg and anti-TPO rate of 54%, and published rates of NSCLC patient pre-treatment rheumatoid factor and antinuclear antibody rates of 28% and 35%, respectively ^39^, are higher than the general population rates of anti-Tg (11%), anti-TPO (13%), rheumatoid factor (5–25%) and antinuclear antibodies (27%) ^48– 50^. While the development of tumor-associated autoantibodies to aberrant protein structures ^12^ has undergone considerable biomarker investigation ^51– 55^, the mechanism of cancer patients developing autoantibodies to endogenous proteins and their biomarker potential is less clear.

The increase in anti-thyroid antibody titers shortly after treatment initiation, or the development of new anti-thyroid antibodies following treatment, points to a possible unmasking of latent autoimmunity by pembrolizumab. Patients with pre-existing latent autoimmunity characterized by increased autoantibody titers may be at higher risk of developing toxicity from ICB. We suggest future prospective studies of other autoimmune markers at baseline are evaluated in patients treated with ICB to further elucidate a mechanism for this effect.

Our study showed that patients can still develop hypothyroidism without the presence of anti-Tg and anti-TPO antibodies. Our reported rate on study of elevated anti-Tg and/or anti-TPO antibodies among those who developed hypothyroidism was 88%, which is comparable to the rates of elevated anti-Tg and anti-TPO antibodies in patients with chronic autoimmune thyroiditis within the general population (70–80% and 90–95%, respectively ^56^). The paucity of anti-Tg and anti-TPO antibodies in those who develop autoimmune primary hypothyroidism implies a non-humoral pathway. As observed in chronic autoimmune thyroiditis, these hypothyroidism events may be secondary to: reduced number and/or function of immune regulatory suppressor cells (e.g. CD4+CD25+) ^57, 58^; the large diversity of the third complementarity-determining region of T-cell surface antigen receptors creating increased propensity of attacking thyroid tissue ^59^; from Th1 cytotoxic T-cell apoptotic destruction of thyroid tissue ^60^; or loss of self-tolerance ^61^ by direct interaction of anti-PD1-/L1 agents with PD-L1/L2 expressed on normal thyroid tissue ^62^. Since Th1 lymphocytes secrete interleukin-2, interferon gamma, and tumor necrosis factor-beta ^63^, evaluation of these cytokines may help elucidate the role of these cells in anti-PD-1-induced non-humoral hypothyroidism.

This study has several limitations. This was a proof-of-concept study designed to screen for candidate autoantibody targets in a mixed solid tumor cohort. Our proteome was not comprehensive and may have missed potential targets during autoantibody candidate selection. During the candidate selection, the status of two patients changed due to the late development of significant toxicity events and four patients developed a toxicity event before the pre-cycle 3 blood draw, possibly skewing these preliminary results. Since the change in peptide quantity was used to select the candidates, the act of pooling serum samples among the four different patient groups may have diluted autoantibody targets present in low titers. Further, the candidates were selected based on peptide number increases from pre- to post-pembrolizumab, thus excluding possible candidates from baseline titers alone. Our study with mixed histologies, each in small numbers, creates a challenge in interpreting the CBR results. In total, 26 patients came off trial prior to pre-cycle 3 blood processing and were removed from the autoantibody analysis. This incomplete patient representation may bias event outcomes and distort the predictive/prognostic potential of the candidate autoantibodies. Since new irAEs from PD1 inhibition can occur many months after starting treatment ^43^, and onset of hypothyroidism post-PD-1 initiation can range from 0.7 weeks to 19 months ^5^, there is the possibility that ongoing patient follow up will yield new toxicity events. This is especially pertinent to the patients who developed non-thyroidal toxicity who have anti-thyroid antibodies present. The small number patients analyzed and limited number of response and toxicity events yields low statistical power and may give spurious results. Ultimately, our findings require further validation in an independent prospective dataset, with adequate sample size to detect histology-specific effects on survival.

## Conclusions

We have shown an association of an increase in anti-Tg and anti-TPO titers between baseline at pre-cycle 3 pembrolizumab with toxicity, elevated pre-cycle 3 anti-Tg with toxicity, and elevated pre-cycle 3 anti-TPO with OS. However, anti-Tg and anti-TPO were unable to predict for non-thyroid toxicity. Future prospective trials evaluating these antibodies, ideally at a time point earlier than pre-cycle 3 of pembrolizumab or with other ICB agents, are needed to validate this finding. Enriching for patients with a single disease site will aid in determining their association with CBR. If successful, this will yield the first data for a minimally invasive, blood-based predictive biomarker to identify which patients derive benefit and/or toxicity from pembrolizumab, sparing unnecessary financial burden and delays to more appropriate care.

## Data availability

### Underlying data

Figshare: Autoantibody dataset.xlsx. https://doi.org/10.6084/m9.figshare.12149598 ^33^.

This file contains the autoantibody dataset generated and assessed during this study, including patient outcomes, autoantibody titers and mass spectrometry data.

### Extended data

Figshare: Extended data.docx. https://doi.org/10.6084/m9.figshare.12149601.v1 ^30^.

This extended data file contains the following information:

- Concentration of protein in various tissue lysates used for autoantibody binding.
- Autoantibody immuno-mass spectrometry methods.
- Immunohistochemistry methods and MPS calculation.
- Beeswarm plots showing anti-Tg antibody titers in those with or without toxicity from pembrolizumab.
- Statistics of anti-Tg and anti-TPO antibody as continuous variables and their association with CBR and toxicity.
- Beeswarm plots showing anti-TPO antibody titers in patients with and without toxicity to pembrolizumab.
- Beeswarm plots showing anti-Tg antibody titers in those with or without CBR from pembrolizumab.
- Beeswarm plots showing anti-TPO antibody titers in those with or without CBR from pembrolizumab.
- Kaplan-Meier PFS analysis using baseline and pre-cycle 3 anti-Tg.
- Kaplan-Meier PFS analysis using baseline and pre-cycle 3 anti-TPO.
- Kaplan-Meier analysis using Δ anti-Tg.
- Kaplan-Meier analysis using Δ anti-TPO.
- Analysis of covariate ethnicity with anti-Tg and anti-TPO titers as a categorical variable.
- Analysis of covariate ethnicity with anti-Tg and anti-TPO titers as continuous variables.
- Analysis of covariate gender with anti-Tg and anti-TPO titers as categorical variables.
- Analysis of covariate gender with anti-Tg and anti-TPO titers as continuous variables.
- Analysis of covariate PD-L1 MPS percentage as a categorical variable with anti-Tg and anti-TPO titers as categorical variables.
- Analysis of covariate PD-L1 MPS percentage as a continuous variable with anti-Tg and anti-TPO titers as categorical variables.
- Analysis of covariate PD-L1 MPS as a categorical variable and anti-Tg and anti-TPO titers as continuous variables.
- Analysis of covariate age as a continuous variable with anti-Tg and anti-TPO titers as categorical variables.
- Analysis of standard prognostic variables age and PD-L1 MPS with anti-Tg and anti-TPO titers, all as continuous variables.

Data are available under the terms of the Creative Commons Attribution 4.0 International license (CC-BY 4.0).
