# Supplementary material for: Predicting response and toxicity to PD-1 inhibition using serum autoantibodies identified from immuno-mass spectrometry
Source: F1000Res. 2020 May 7;9:337. [Version 1] doi: 10.12688/f1000research.22715.1 (PMC7707117; doi:10.12688/f1000research.22715.1)
Supplement: Peer review history

# Peer review: Reviewer response for version 1

**DOI:** 10.5256/f1000research.25082.r75094

**Published:** 2020-11-30

**Authors:** Francesco Salvatore, Domenico Salvatore, Porcelli Tommaso

**Affiliations:** 1 CEINGE-Biotecnologie Avanzate s.c.ar.l,, Naples, Italy; 2 Chair of Endocrinology, School of Medicine, University of Naples Federico II, Naples, Italy

The aim of this work by Music *et al*. was to identify biomarkers able to predict the clinical benefit and the risk of irAEs in patients treated with pembrolizumab. In a discovery analysis, the authors found anti-Tg Ab to be significantly increased by pembrolizumab. Therefore, anti-thyroid Ab (i.e., anti-Tg and anti-TPO Abs) were evaluated as predictive biomarkers of tumor response and treatment toxicity in 78 patients treated with pembrolizumab for different tumor types. The aim of the paper appears highly interesting. The authors clearly show that patients with an increase in baseline anti-Tg and anti-TPO titers after 7 weeks of treatment presented higher rate of toxicity in response to pembrolizumab. Moreover, the authors found an association between anti-TPO titer and overall survival, although without benefit in progression-free survival.

 Interestingly, 50% of patients that developed hypothyroidism as irAEs were treated with pembrolizumab for a melanoma cancer (i.e. 14% of enrolled patients). Moreover, as shown in table 1, the melanoma group presented the highest rate of patients with clinical benefit rate (CBR) (8/11, 73%) and with toxicity (6/11, 55%). Conversely, other cancers showed very different responses. For example, the triple negative breast cancer presented no cases of hypothyroidism, a low CBR (1/12, 8%) and a low toxicity (1/12, 8%). Accordingly, 6/11 of melanoma patients presented baseline thyroid-Ab alterations and 4/11 presented increase in thyroid-Ab after treatment. I would have suggested to comment in the discussion the strong association between anti-thyroid Ab and CBR/ toxicity in melanoma patients.

 The cutoff to define auto-Ab elevation was set at 10.0 IU/ml. As shown in the “Autoantibody dataset” tables (ref 33), in 4 patients considered with elevated basal Tg-Ab, the Ab titer was <10.9 IU/ml (in one case was 10.06 IU/ml) and in 3 of these 4 patients there was no increase at pre-cycle 3 measurement. Similarly, 4 patients from the group with elevated pre-cycle 3 TPO-Ab presented only slightly elevated values (in 2 cases, 10.02 UI/ml). I’m not sure that values of 10.02 IU/ml can be considered as positive, especially if no increase (or a decrease) was observed after treatment. This is important because the reported association between overall survival and elevated pre-cycle 3 anti-TPO Ab is based on these values. I would have suggested to specify in the “Methods” section if serum anti-Ab measurement were repeated for confirmation and if the reported values are a mean value of more measurements.

 In Figure 1, in the “outcomes” boxes, the reasons for stopping treatment include both clinical progression and RECIST progression. I would have specified in the “Methods” section which are the criteria for the definition of “clinical progression” and when/why this parameter is chosen instead of RECIST progression to decide for the treatment stop.

In conclusion this is an highly interesting paper, the topic of this study is interesting and the work provides new insight into the field. This proof of concept paper has the additional merit to allow other cohorts of patients to be enrolled and therefore extend the casistics.

Is the work clearly and accurately presented and does it cite the current literature?

Yes

If applicable, is the statistical analysis and its interpretation appropriate?

Yes

Are all the source data underlying the results available to ensure full reproducibility?

Yes

Is the study design appropriate and is the work technically sound?

Yes

Are the conclusions drawn adequately supported by the results?

Yes

Are sufficient details of methods and analysis provided to allow replication by others?

Yes

Reviewer Expertise:

Reviewer Francesco Salvatore: pathophysiology and diseases; Reviewers Tommaso Porcelli and Domenico Salvatore: thyroid physiology

We confirm that we have read this submission and believe that we have an appropriate level of expertise to confirm that it is of an acceptable scientific standard.

# Peer review: Reviewer response for version 1

**DOI:** 10.5256/f1000research.25082.r69349

**Published:** 2020-09-07

**Authors:** Gerasimos Sykiotis

**Affiliations:** 1 Service of Endocrinology, Diabetology and Metabolism, Lausanne University Hospital & University of Lausanne, Lausanne, Switzerland

The manuscript if fully well summarized in its abstract, hence there is no need to provide a separate summary here. I recommend to revise the text to address the following comments:

1. The main irAE in the cohort was hypothyroidism. It is then not surprising that the main autoantibodies identified were thyroid-specific proteins, and actually they were “the usual suspects”. In the Discussion, it is mentioned that new insights might be obtained by analyzing cohorts homogeneous as to the primary tumor site. But is there any evidence that the irAE profile depends on the primary tumor site? If so, then it should be cited in support. If not, then this statement should be revised. In either case, it may be more productive to profile (sub)cohorts homogeneous as to the type of irAE(s), rather than the primary tumor site.
2.  On the one hand, the analyses shown in Table 2 show no association between anti-Tg antibody levels at baseline and toxicity. On the other hand, the first paragraph on page 9 mentions that hypothyroidism development during treatment was more common among patients with elevated anti-Tg titers at baseline. These two statements appear contradictory, and I would recommend to address and explain this directly in the Discussion. For example, is it because the “toxicity” assessed in Table 2 includes all irAEs?
3. In the second paragraph of page 9, it is mentioned that 60% of patients who did not develop hypothyroidism had elevated anti-Tg or anti-TPO antibody titers at baseline and/or pre-cycle 3. This % is quite high. I recommend that the authors mention specifically the % of patients with positive antibodies at baseline (this is actually done in the Discussion, but for the overall cohort, not specifically for the patients who did not develop hypothyroidism). Also, statistics aside, it appears intuitive to a clinician that if the % of positivity at baseline is so high among patients who do not develop hypothyroidism during treatment, then the predictive value of this baseline assessment will be very low for any given individual patient. I think that this merits a mention.
4.  I recommend to address whether patients with positive/elevated anti-Tg or anti-TPO antibodies at baseline were more likely to show an increase of these titers during treatment.
5.  Were any patients with pre-existing thyroid disease included in the discovery cohort (top of text in page 9)?

Is the work clearly and accurately presented and does it cite the current literature?

Yes

If applicable, is the statistical analysis and its interpretation appropriate?

Yes

Are all the source data underlying the results available to ensure full reproducibility?

Yes

Is the study design appropriate and is the work technically sound?

Yes

Are the conclusions drawn adequately supported by the results?

Partly

Are sufficient details of methods and analysis provided to allow replication by others?

Yes

Reviewer Expertise:

Thyroid pyhsiology, pathophysiology and disease.

I confirm that I have read this submission and believe that I have an appropriate level of expertise to confirm that it is of an acceptable scientific standard.
